# Photopharmacology: A new paradigm for vision restoration

**DOI:** 10.1002/ctm2.70779

**Published:** 2026-08-13

**Authors:** Robert J. Casson

**Affiliations:** ^1^ Department of Ophthalmology Royal Adelaide Hospital Adelaide South Australia Australia; ^2^ Discipline of Ophthalmology & Visual Sciences, Adelaide Adelaide University South Australia Australia

1

Inherited retinal diseases (IRDs) comprise a diverse group of genetic disorders characterized by progressive degeneration of photoreceptors, ultimately leading to irreversible vision loss and blindness. Among IRDs, retinitis pigmentosa (RP) is one of the most common causes of blindness in the working‐age population in high‐income countries, with an estimated prevalence of approximately 1 in 3000–4000 individuals.^1^, ^2^, ^3^ The approval of voretigene neparvovec (Luxturna) for RPE65‐associated retinal dystrophy established a landmark proof‐of‐principle for gene replacement therapy in IRD. However, current gene therapy strategies generally remain mutation‐specific and require sufficient survival of functional photoreceptors. As degeneration progresses and photoreceptors are lost, the therapeutic challenge shifts from preserving vision to restoring it. This unmet need has driven the development of gene‐agnostic restorative strategies that seek to bypass or replace the damaged outer retina, including electronic retinal prostheses, optogenetic therapies, cell‐based approaches and, more recently, chemical photoswitches.^4^

Chemical photoswitches represent a distinctive pharmacological approach to vision restoration. These light‐responsive small molecules reversibly modulate neuronal ion channels, enabling surviving inner retinal neurons to respond to light and engage downstream visual pathways.^5^ Evidence from preclinical studies suggests that KIO‐301 selectively associates with hyperpolarization‐activated cyclic nucleotide‐gated channels expressed by retinal ganglion cells.^6^ Light‐induced conformational changes in the molecule modulate channel activity, conferring light responsiveness to a subclass of retinal ganglion cells.^6^ In effect, these retinal ganglion cells function as surrogate photoreceptors, transmitting light‐evoked signals through the optic nerve to higher visual centres and thereby providing the neural substrate for visual perception. The proposed mechanism of action is shown in Figure 1. Unlike prosthetic, gene‐based or cell‐based interventions, photopharmacology does not require implanted hardware, gene transfer or cellular replacement. Instead, it exploits surviving inner retinal circuitry through intravitreal administration of a small molecule, offering the potential for mutation‐agnostic treatment, repeat dosing and iterative optimisation through medicinal chemistry. These features also confer several practical advantages over existing restorative approaches. As a small‐molecule therapy, photoswitches can be manufactured using established pharmaceutical processes, administered using routine intravitreal injection techniques familiar to ophthalmologists and refined through successive generations of medicinal chemistry. This pharmacological flexibility provides opportunities to optimise potency, spectral sensitivity, duration of action and retinal selectivity as clinical experience evolves. These characteristics laid the foundation for the first clinical evaluation of an intravitreal chemical photoswitch in patients with advanced RP.

**FIGURE 1:**
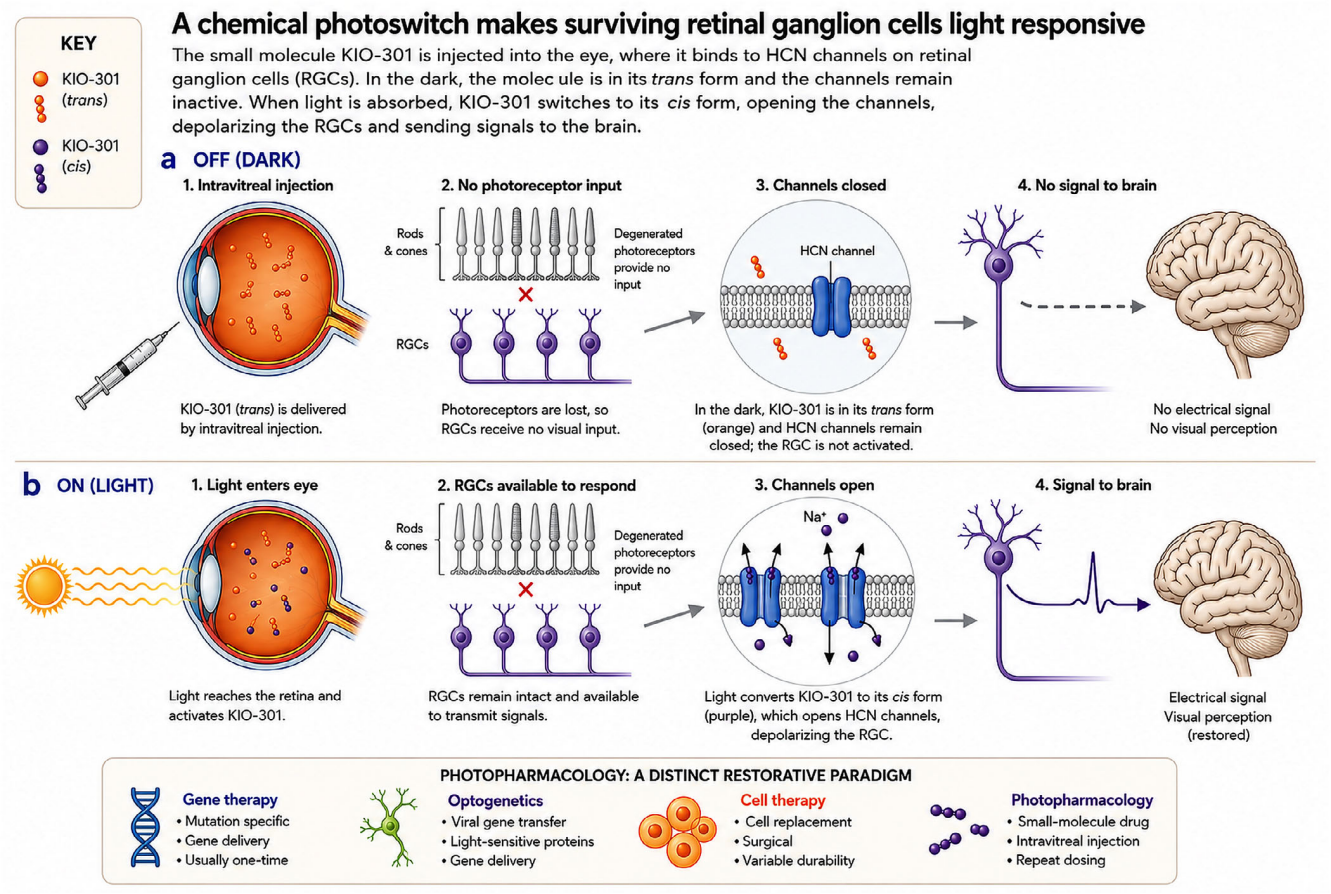
Conceptual model of photoswitch‐mediated retinal ganglion cell photosensitization. This schematic illustrates a hypothesis‐generating conceptual framework, informed by prior preclinical studies of azobenzene photoswitches, for how KIO‐301 may confer light responsiveness to retinal ganglion cells (RGCs) in the setting of advanced photoreceptor degeneration.

The ABACUS‐1 clinical trial was designed to evaluate the safety, tolerability and preliminary biological activity of KIO‐301, the first intravitreally administered chemical photoswitch to enter human clinical testing.^7^ Patients with advanced RP were deliberately selected because severe photoreceptor loss leaves few therapeutic alternatives while preserving much of the downstream retinal circuitry that photoswitches seek to engage. In this first‐in‐human, open‐label Phase I study, KIO‐301 demonstrated a favourable safety profile, with no serious adverse events or drug‐related intraocular inflammation.^7^ This finding is particularly encouraging given that intraocular inflammation remains an important consideration for several retinal gene therapy platforms, reflecting the biological challenges associated with viral vector delivery and sustained transgene expression. As a non‐viral, small‐molecule therapy delivered by routine intravitreal injection, photopharmacology may offer a fundamentally different safety paradigm. Importantly, repeated intravitreal administration is already well established in modern ophthalmic practice through the widespread use of anti‐vascular endothelial growth factor therapies, making repeat dosing a familiar and clinically practical treatment strategy. Although larger studies with longer follow‐up are required, the absence of significant inflammatory toxicity in ABACUS‐1 supports the feasibility of repeated pharmacological administration. Although not powered to establish efficacy, exploratory assessments identified encouraging signals of pharmacodynamic activity, including improvements in selected visual function measures and light‐evoked cortical activation on functional MRI, providing independent evidence that pharmacological restoration of retinal light responsiveness can engage the human visual pathway. First‐in‐human studies necessarily prioritize safety over efficacy, and the trial was neither designed nor powered to demonstrate clinically meaningful improvements in vision. Nevertheless, the convergence of favourable safety findings with objective evidence of cortical activation provides an important translational bridge between decades of preclinical research and subsequent efficacy‐focused clinical trials.

The initiation of the multicentre, randomised, placebo‐controlled Phase II trial (ABACUS II) represents the first opportunity to rigorously determine whether these encouraging pharmacodynamic signals translate into clinically meaningful and sustained improvements in visual function. Beyond confirming safety over a longer treatment period, controlled evaluation will be essential to define the magnitude, durability and clinical relevance of any treatment effect. Beyond KIO‐301, continued advances in molecular design, retinal targeting and pharmacokinetics are likely to expand the therapeutic potential of chemical photoswitches.

More broadly, photopharmacology illustrates the emergence of pharmacological vision restoration as a new therapeutic paradigm. Rather than preserving remaining photoreceptors or permanently modifying retinal biology, chemical photoswitches seek to reprogram surviving neural circuits to restore light responsiveness. Whether photopharmacology ultimately occupies a complementary or distinct place alongside gene therapy, optogenetics and cell replacement will depend on future clinical studies. Nevertheless, the successful translation of a chemical photoswitch into human clinical trials represents an important milestone, demonstrating that vision restoration through pharmacological manipulation of surviving retinal circuitry is no longer purely a theoretical concept.

## CONFLICT OF INTEREST STATEMENT

The author declares no conflicts of interest.
